# Nutritional Supplementation Is a Necessary Complement to Dietary Counseling among Tuberculosis and Tuberculosis-HIV Patients

**DOI:** 10.1371/journal.pone.0134785

**Published:** 2015-08-27

**Authors:** Adriana Costa Bacelo, Andrea Ramalho, Pedro Emmanuel Brasil, Cláudia dos Santos Cople-Rodrigues, Ingebourg Georg, Eliane Paiva, Sheila Vasques Leandro Argolo, Valeria Cavalcante Rolla

**Affiliations:** 1 Clinical Research Laboratory on Mycobacteria, National Institute of Infectious Diseases Evandro Chagas, Fiocruz, Rio de Janeiro/RJ, Brazil; 2 Nutrition Service, National Institute of Infectious Diseases Evandro Chagas, Fiocruz, Rio de Janeiro/RJ, Brazil; 3 Josué de Castro Institute, UFRJ, Rio de Janeiro/RJ, Brazil; 4 Clinical Reasearch Laboratory on Chagas Disease, National Institute of Infectious Diseases Evandro Chagas, Fiocruz, Rio de Janeiro/RJ, Brazil; 5 Institute of Nutrition, UERJ, Rio de Janeiro/RJ, Brazil; 6 Diagnostics Activities Coordinating, Immunodiagnostic Section, National Institute of Infectious Diseases Evandro Chagas, Fiocruz, Rio de Janeiro/RJ, Brazil; 7 Department of Nutrition, UNISUAM, Rio de Janeiro/RJ, Brazil; 8 Sergio Franco Laboratory, Caxias/RJ, Brazil; Temple University School of Medicine, UNITED STATES

## Abstract

The Brazilian Ministry of Health and the World Health Organization recommend dietary counseling for patients with malnutrition during tuberculosis treatment. Patients under tuberculosis therapy (infected and not infected with HIV) were followed-up to evaluate the effectiveness of dietary counseling. **Objective:** describe the nutritional status of patients with tuberculosis. Methods: an observational follow-up study over a 180-day period of tuberculosis therapy in adults was conducted. Subjects were assessed for body composition (using BMI, TSF and MUAC parameters), serum biomarkers and offered dietary counseling. The data obtained at each visit (D15, D30, D60, D90, D120, D150, and D180) were analyzed, showing trajectories over time and central tendencies each time. **Results:** at baseline, the mean age was 41.1 (±13.4) years; they were predominantly male, with income lower than a local minimum wage and at least six years of schooling. Patients showed predominantly pulmonary tuberculosis. At baseline, all patients suffered from malnutrition. The overall energy malnutrition prevalence was of 70.6%. Anemia at baseline was observed in both groups (63.2%), however, it was significantly more pronounced in the HIV+. At the end, energy malnutrition was reduced to 57.1% (42.9% of HIV- and 71.4% of the HIV+). Micronutrients malnutrition was evident in 71.4% of the HIV- patients and 85.7% of HIV+ patients at the end of tuberculosis therapy. Using BMI (≤18.5 kg/m^2^cutoff) as an index of malnutrition, it was detected in 23.9% of the HIV- and 27.3% of the HIV+ patients at baseline, with no evident improvement over time; using TSF (≤11.4mm as cutoff) or MUAC (≤28.5cm as cutoff), malnutrition was detected in 70.1% and 85.3% of all patients, respectively. Nevertheless, combining all biomarkers, at the end of follow-up, all patients suffered from malnutrition. **Conclusion:** Although with a limited number of patients, the evidence does not support that dietary counseling is effective to recover from malnutrition in our population.

## Introduction

Tuberculosis and the human immunodeficiency virus (HIV) are historically associated with malnutrition, reduced appetite, low dietary intake, malabsorption and increased caloric demand [1–4].

The Brazilian Ministry of Health (BMH) [5] and the World Health Organization (WHO) [6] recognize the existence of caloric malnutrition, protein malnutrition and micronutrient deficits associated with tuberculosis and HIV infection. This is followed by the recommendation that patients with mild or moderate malnutrition should eat a healthy diet that meets the Recommended Dietary Allowance (RDA) [7,8]. On the other hand, patients with severe malnutrition should have food supplementation. However, neither BMH nor WHO currently recommend any specific nutritional supplementation for severe malnutrition in these settings.

Studies conducted with patients being treated for tuberculosis, who followed dietary advice exclusively or received supplemental food donation [9–11], showed early BMI improvement. Additional parameters with evidence of improvement with this intervention were: total caloric intake, sanitary requirements for food preparation, serum protein and clinical response. This intervention also determined earlier improvement on acid-fast bacilli detection on sputum smears and reduced bronchial secretion [9].

There is an extensive discussion on the nutritional status of patients with tuberculosis, including the occurrence of anemia, reduced food consumption and adherence to treatment when food is donated [12], but not much has been studied on nutritional supplementation among brazilians [13–17]. The discussion of nutritional status before, during and after tuberculosis treatment in patients treated in Brazil is particularly intriguing, as the brazilian health care system provides free access to tuberculosis and HIV treatments. This is a unique situation, and it is not expected that findings from studies conducted in other countries [3,4,11,18,19] could be extrapolated to brazilian patients.

This study aims to describe the nutritional status of patients with tuberculosis, including those infected with HIV, who received dietary counseling exclusively during tuberculosis treatment in Rio de Janeiro, and to evaluate if this strategy is enough to enhance recovery from their nutritional impairment observed at the beginning of tuberculosis treatment.

## Methods

### Study design

This is an observational prospective follow-up study that was conducted in patients who received tuberculosis diagnosis from July 2008 to September 2013.

### Eligibility and ineligibility criteria

Patients were eligible if they had tuberculosis, whether infected or not with HIV, and if they were between 18 and 65 years old and agreed to participate in the study. HIV positive patients were naïve of antiretrovirals (ARV). Patients were urged to refrain from using nutritional supplements during the entire study period. Exclusion criteria were: those who had hypermetabolism, digestive diseases or who used any nutritional supplementation up to six months before tuberculosis treatment. Only the supplementation of pyridoxine (vitamin B6) was allowed, in conformity with the protocol of the Brazilian Ministry of Health [20], which recommends vitamin B6 concomitant with isoniazid for all patients being treated for tuberculosis. Individuals were discontinued from the study if they presented severe malnutrition at any point, as in such cases a supplementation was provided.

### Clinical diagnosis and nutrition

The diagnosis of tuberculosis was established by assessing clinical signs and symptoms, compatible radiological findings and the identification of *Mycobacterium tuberculosis* in culture. In cases where the *Mycobacterium tuberculosis* was not isolated, the diagnosis of tuberculosis was made using therapeutic response [20]. The strategies for both tuberculosis and HIV infection diagnosis and treatment followed the Brazilian Ministry of Health recommendations [5,5,21], which recommend a 4 drugs combination for tuberculosis (rifampicin, isoniazid, ethambutol and pyrazinamide) and efavirenz based regimen for HIV/AIDS.

**Fig 1:**
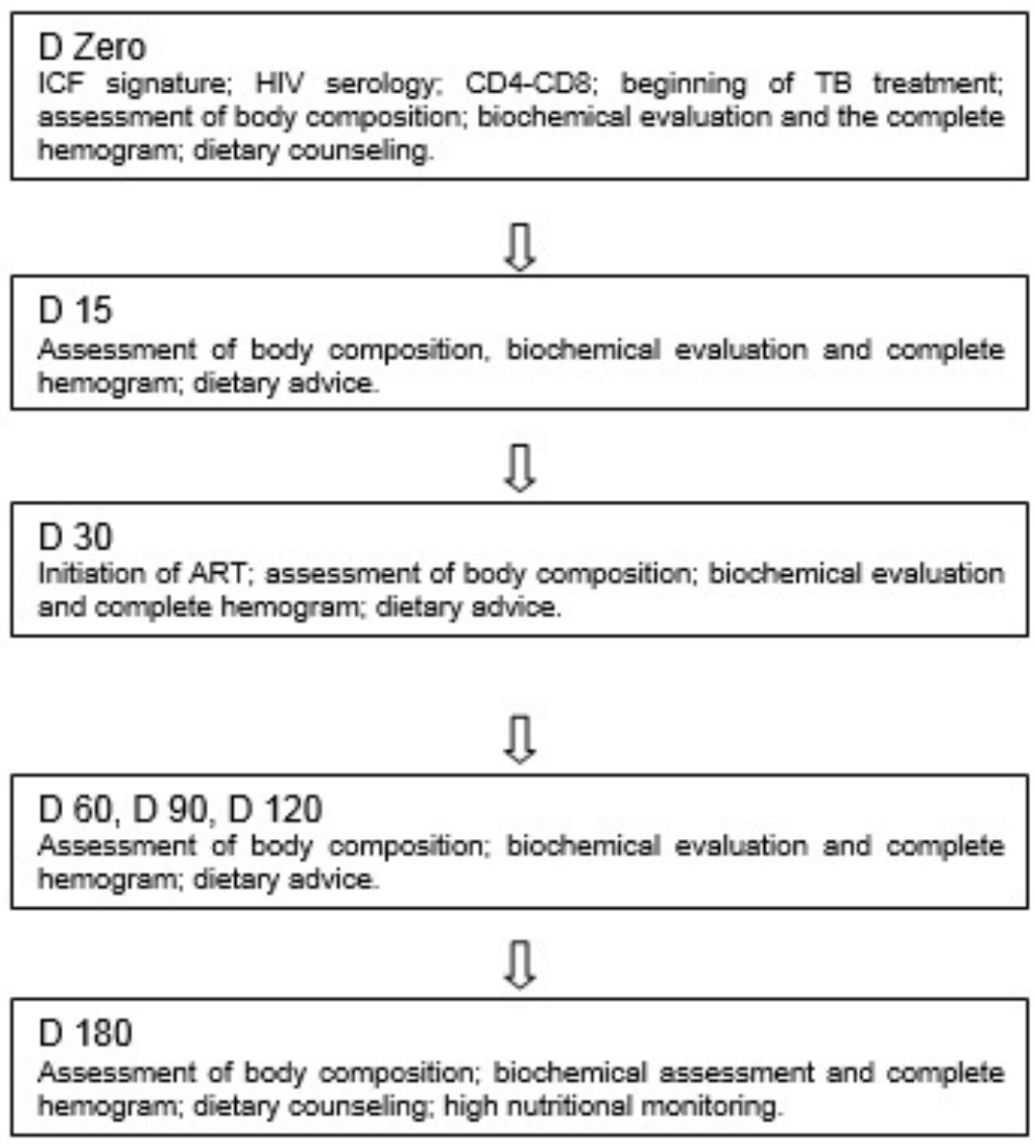
Flowchart of the study procedures. D = day of visits; ICF = informed consent form; HIV = human immununodeficiency virus; ART = antiretroviral therapy; Biochemical assessment = albumin, iron fixation capacity, iron, selenium, zinc, retinol and tocopherol; CD4 = lymphocyte TCD4; CD8 = lymphocyte TCD8; VL = viral load; dietary counselling = was delivered and read with patients the document proposed by the ministry of health (“CGAN—Coordenação-Geral de Alimentação e Nutrição” 2014).

Patients underwent nutritional evaluation and received dietary counseling after tuberculosis diagnosis and during their tuberculosis treatment. The steps and procedures used in this study are presented in Fig 1.

Anthropometric and biochemical biomarkers, including the analysis of serum micronutrients, were used to assess nutritional status. Anthropometric evaluation was conducted using the body mass index (BMI– 18.5–24.9Kg/m^2^), mid–upper-arm circumference (MUAC– 28.5–29.3cm), triceps skinfold (TSF– 11.4–18.2mm) and mid-arm muscle circumference (MAMC– 21.0–27.8cm), according to Lohman [22] (Table 1).

**Table 1:** Measurements of body composition and biomarkers of all patients throughout tuberculosis treatment.

Time course of tuberculosis treatment (days)									
	RV	D0	D15	D30	D60	D90	D120	D150	D180
Current weight^1^ ^,^ ^2^	-	58.2 (±11.8)	60.1 (±14.1)	59.5 (±12.2)	60.4 (±11.7)	61.2 (±14.9)	66.6 (±13.9)	61.8 (±9.8)	62.0 (±10.1)
BMI^1^ ^,^ ^3^	18.5–24.9	21.0 (±3.9)	21.1 (±4.1)	21.1 (±3.4)	22.0 (±3.4)	21.7 (±4.3)	23.0 (±4.1)	22.5 (±3.3)	21.8 (±2.8)
MUAC^1^ ^,^ ^4^	28.5–29.3	25.4 (±3.8)	26.2 (±4.1)	26.3 (±3.6)	26.9 (±3.4)	26.4 (±5.0)	27.2 (±2.8)	27.2 (±2.7)	27.5 (±2.9)
TSF^1^ ^,^ ^5^	11.4–18.2	11.9 (±7.5)	13.5 (±11.9)	12.3 (±8.4)	19.5 (±11.4)	15.8 (±9.8)	13.2 (±7.3)	13.3 (±6.6)	12.9 (±5.1)
MAMC^1^ ^,^ ^4^	21.0–27.8	21.4 (±4.0)	21.6 (±3.1)	22.1 (±3.1)	21.1 (±3.8)	21.8 (±4.1)	23.1 (±2.3)	23.2 (±2.2)	23.7 (±2.8)
Hemoglobin^1^ ^,^ ^6^	11.0–18.0	11.8 (±2.0)	12.1 (±1.6)	12.3 (±1.9)	12.5 (±1.6)	12.4 (±1.9)	13.9 (±1.4)	12.6 (±1.6)	13.4 (±1.3)
Hematocrit^1^ ^,^ ^7^	34.0–54.0	35.7 (±5.7)	35.8 (±4.4)	36.7 (±5.3)	37.9 (±4.3)	37.1 (±5.5)	40.6 (±3.7)	37.8 (±4.6)	38.0 (±3.5)
Globulin ^1^ ^,^ ^6^	2.5–4.0	5.2 (±1.5)	4.5 (±1.5)	4.6 (±1.6)	3.8 (±0.6)	5.1 (±1.9)	4.5 (±1.4)	5.3 (±1.2)	4.4 (±1.0)
Albumin ^1^ ^,^ ^6^	3.4–5.0	3.1 (±0.7)	3.2 (±0.7)	3.3 (±0.6)	3.5 (±0.6)	3.4 (±0.6)	3.7 (±0.3)	3.5 (±0.6)	3.7 (±0.5)
TIBC^1^ ^,^ ^8^	250.0–450.0	228.7 (±49.0)	237.1 (±63.5)	252.0 (±61.8)	236.0 (±38.5)	239.4 (±62.6)	274.3 (±64.9)	284.8 (±60.7)	253.0 (±47.8)
Transferrin ^1^ ^,^ ^9^	250.0–300.0	139.9 (±39.2)	158.9 (±50.1)	159.6 (±49.6)	145.8 (±30.8)	155.8 (±38.8)	177.1 (±53.0)	193.7 (±41.2)	152.5 (±40.0)
Retinol ^1^ ^,^ ^8^	>0.7	0.4 (±0.2)	0.3 (±0.1)	0.4 (±0.2)	0.3 (±0.2)	0.4 (±0.1)	0.5 (±0.2)	0.4 (±0.2)	0.5 (±0.2)
Tocopherol^1^ ^,^ ^10^	>5.0	8.4 (±4.4)	9.0 (±3.5)	9.3 (±2.8)	10.6 (±4.4)	9.1 (±2.0)	9.6 (±1.9)	9.5 (±2.1)	9.6 (±5.2)
Zinc ^1^ ^,^ ^9^	> 70.0	80.3 (±20.5)	71.8 (±12.5)	102.8 (±121.1)	75.6 (±20.0)	89.3 (±27.2)	94.9 (±22.1)	218.3 (±389.4)	85.3 (±24.3)
Selenium^1^ ^,^ ^11^	70.0–90.0	73.8 (±50.1)	66.0 (±19.8)	76.7 (±38.4)	90.3 (±56.3)	49.8 (±18.5)	79.6 (±40.5)	60.8 (±22.4)	65.9 (±27.4)
Iron^1^ ^,^ ^8^	50.0–170.0	53.1 (±36.0)	60.5 (±33.8)	61.3 (±33.4)	65.0 (±30.2)	66.2 (±30.9)	71.9 (±23.6)	76.1 (±23.7)	76.2 (±29.0)

1 = mean (SD)

2 = kilogram (Kg)

3 = kilogram per square meter (Kg/m^2^)

4 = centimeters (cm)

5 = millimeters (mm)

6 = grams per deciliter (g/dl)

7 = percentage (%)

8 = micrograms per deciliter (mcg/dl)

9 = milligrams per deciliter (mg/dl)

10 = milligrams per liter (mg/l)

11 = micrograms per liter (mcg/l)

BMI = body mass index; MUAC = mid–upper-arm circumference; MAMC = mid arm muscle circumference; TSF = triceps skinfold thickness; TIBIC = iron fixation capacity; RV = reference values.

Serum biomarkers were assessed, including analysis of blood count, using automated optical microscopy with reference value of 11–18 (g/dl) for hemoglobin and 34–54 (%) for hematocrit. Chromatic measurement with a reference value of 3.4–5.0 for albumin; 6.4–8.2 (g/dl) for total protein; 2.5–4.0 (g/dl) for globulin; 250–450 (mcg/dl) for iron fixation capacity. High-performance liquid chromatography with a reference value of >0.7 (mcg/dl) for retinol and >0.5 (mg/l) for tocopherol. Atomic absorption with reference value of >70 (mg/dl) for zinc, 70–90 (mcg/l) for selenium and 50–170 (mcg/dl) for iron (Table 1).

Serum samples were frozen (-20°C to -10°C) for analysis of vitamins A, E and zinc; and plasma was refrigerated from 2 to 8°C for selenium analysis. All tubes were transported to the laboratory for analysis, with a maximum of 7 days of storage. The analyses for the remaining biomarkers were done immediately after blood collection.

The study endpoint was the recovery from malnutrition. Individuals were considered to be without malnutrition, at any time, if all the biomarkers (anthropometric, hematological, biochemical and micronutrients) were within the reference values, as established by FAO [23]. And they were considered to be malnourished, at any time, if at least one of the biomarkers was outside the reference values, as established by FAO [23].

Patients were classified within the following malnutrition statuses, at every moment: (a) global, using BMI, TSF, MUAC, serum protein, albumin, transferrin and iron fixation; (b) energy, using the parameters BMI and TSF; (c) protein, using the somatic parameter MAMC, and visceral parameters serum protein, albumin, transferrin and iron fixation; (d) micronutrients, using serum biomarkers (iron, selenium, zinc, retinol and tocopherol).

### Dietary counseling

The dietary counseling proposed by BMH [24] was delivered at every single visit, to every participant. At each visit, participants had time to ask questions, clear up any doubts about their diet and to correct some identified misconceptions.

### Ethical Aspects

This study was conducted in accordance with the ethical standards outlined for research in humans in the declaration of Helsinki, and approved by the Research Ethics Committee of the National Institute of Infectious Diseases Evandro Chagas, under identification number CAE 0008.0.009.000–08. All patients who agreed to participate in the study signed an informed consent form.

### Analysis Plan

The data were analyzed using the R-project software, version 3.0.2. The results were obtained by dividing the data into a baseline time point and follow-up (S1 Dataset). For baseline, the fractions and number of patients in each category were shown for categorical variables. For the continuous variables, either the mean or standard deviation, if the variable follows the normal distribution or median and interquartile range, were estimated. At follow-up, the same information was shown, but stratified by time. The graphs show bootstrap correlation adjusted trajectories for each variable, or the fraction of subjects with malnutrition at each time. Hypothesis tests were considered statistically significant when p ≤ 0.05.

## Results

**Fig 2:**
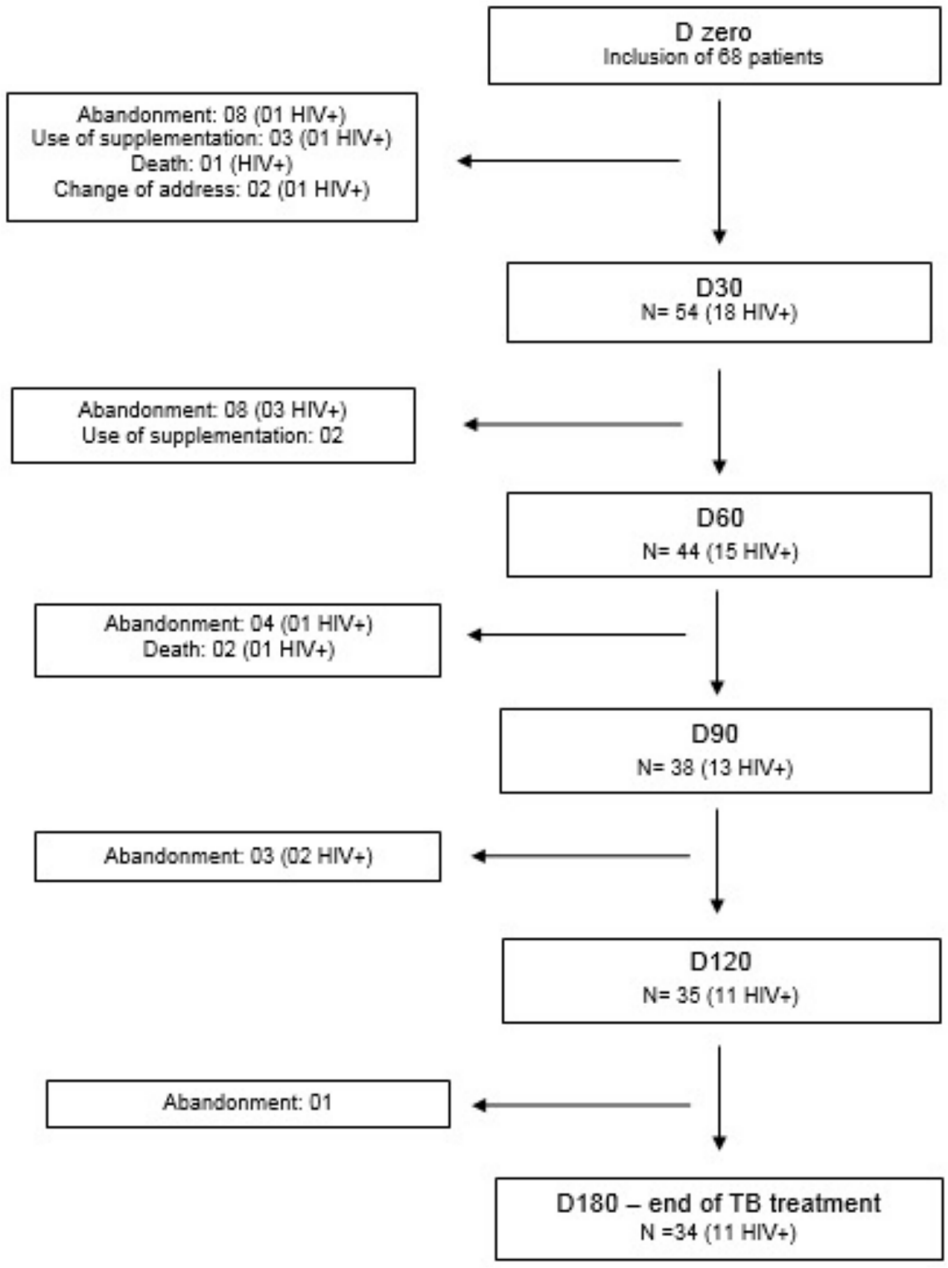
Flowchart with causes of discontinuation throughout the TB treatment course. HIV+ = human immununodeficiency virus infected patients.

Sixty-eight patients were included in the study. 22 were HIV positive and 46 were HIV negative. Exclusions and their reasons were: 5 patients needed nutritional supplementation, 3 patients died and 24 patients were lost to follow-up. Thirty four patients remained until the end of the study (Fig 2).

At baseline the mean (standard deviation) age was 41.1 (±13.4) years old and there was a predominance of males, with income below a local minimum wage, average of six years of education, and pulmonary clinical form of tuberculosis (Table 2). In addition, a minority of patients were drinkers and smokers. Twenty-two of the volunteers were not infected with HIV (Table 2). HIV-positive patients were naïve of antiretrovirals and received efavirenz based regimens 30 days after tuberculosis therapy introduction, as recommended by brazilian guidelines at that time [21].

**Table 2:** Socio-demographic and clinical characteristics of individuals with tuberculosis, who are either HIV-infected or non-infected at the beginning of the follow-up.

	HIV -		HIV +	Total	Hypothesis testing	P value
Total	46		22	68		
**Sex** ^**1**^					Chisq. (1 df) = 1.04	0.308
Male	26 (56.5)		16 (72.7)	42 (61.8)		
Female	20 (43.5)		6 (27.3)	26 (38.2)		
**Age**					t-test (66 df) = 0.62	0.535
Mean (SD)	40.4 (15.1)		42.6 (9.1)	41.1 (13.4)		
**Age groups** ^**1**^ ^,^ ^2^					Fisher's exact test	0.043
16–24	7 (15.2)		1 (4.5)	8 (11.8)		
25–34	15 (32.6)		3 (13.6)	18 (26.5)		
35–44	6 (13.0)		9 (40.9)	15 (22.1)		
45–54	8 (17.4)		7 (31.8)	15 (22.1)		
55–64	8 (17.4)		2 (9.1)	10 (14.7)		
65–71	2 (4.3)		0 (0.0)	2 (2.9)		
**Education**					Rank-Sum test	0.961
Median (IQR)	6 (4.0–1.0)		5 (4.0–9.0)	6 (4.0–10.8)		
**Monthly income** ^3^					Rank-Sum test	0.666
Median (IQR)	800 (550.0–1244.0)		800 (620.5–1560.0)	800 (585.0–1370.0)		
**Smoker** ^**1**^					Chisq. (2 df) = 1.8	0.407
No	25 (54.3)		14 (63.6)	39 (57.4)		
Yes	8 (17.4)		5 (22.7)	13 (19.1)		
Ex-smoker	13 (28.3)		3 (13.6)	16 (23.5)		
**Alcoholism** ^**1**^					Chisq. (2 df) = 2.02	0.365
No	23 (50.0)		12 (54.5)	35 (51.5)		
Yes	13 (28.3)		3 (13.6)	16 (23.5)		
Ex-alcoholic	10 (21.7)		7 (31.8)	17 (25.0)		
**Clinical form of tuberculosis** ^**1**^					Fisher's exact test	0.218
Pulmonary		15 (71.4)	5 (38.5)	20 (58.8)		
Extra pulmonary		4 (19.0)	5 (38.5)	9 (26.5)		
Disseminated		2 (9.5)	3 (23.1)	5 (14.7)		

^1^ = n (%)

^2^ = years

^3^ = Real (R$)

IQR = interquartile range.

At the beginning of the study, all of the patients exhibited malnutrition (Table 3). In both HIV-infected and non-infected patients, micronutrient malnutrition, reduced visceral and somatic protein levels and energy malnutrition were found. However, total protein levels, hemoglobin and hematocrit had a more intense impairment among those infected with HIV (Table 3).

**Table 3:** Biochemical and hematological assessment of HIV-infected and non-infected patients at the beginning of the follow-up.

	HIV -	HIV +	Total	Teste de hypotheses	P valor
**Total**	46	22	68		
**Arm circumference**				Rank-Sum test	0.506
Median (IQR)	25 (23.0–27.0)	24 (23.0–26.0)	25 (23.0–27.0)		
**MUAC classification** ^**1**^				Fisher's exact test	1.000
Below normal	39 (84.8)	19 (86.4)	58 (85.3)		
Normal	6 (13,0)	3 (13.6)	9 (13.2)		
No information available	1 (2.2)	0 (0.0)	1 (1.5)		
**Triceps**				Rank-Sum test	0.444
Median (IQR)	10 (6.0–16.0)	10 (6.2–11.0)	10 (6.0–15.0)		
**TSF classification** ^**1**^				Fisher's exact test	0.296
Below normal	29 (64.4)	18 (81.8)	47 (70.1)		
Normal	5 (11.1)	2 (9.1)	7 (10.4)		
Above	11 (24.4)	2 (9.1)	13 (19.4)		
**Body mass index**				Rank-Sum test	0.322
Median (IQR)	20.6 (18.5–22.9)	19.7 (18–21.1)	20.5 (18.5–22.4)		
**BMI classification** ^**1**^				Chisq. (2 df) = 0.51	0.776
Below normal	11 (23.9)	6 (27.3)	17 (25.0)		
Normal	28 (60.9)	14 (63.6)	42 (61.8)		
Above	7 (15.2)	2 (9.1)	9 (13.2)		
**Energetic nutrition** ^**1**^				Fisher's exact test	0.422
Malnutrition	30 (65.2)	18 (81.8)	48 (70.6)		
Normal	15 (32.6)	4 (18.2)	19 (27.9)		
No information available	1 (2.2)	0 (0.0)	1 (1.5)		
**Mid-arm muscle circumference**				Rank-Sum test	0.758
Median(IQR)	21.1 (19.8–23.4)	21.4 (20.2–23.2)	21.2 (19.9–23.2)		
**MAMC classification** ^1^				Fisher's exact test	0.713
Below normal	29 (63.0)	15 (68.2)	44 (64.7)		
Normal	16 (34.8)	6 (27.3)	22 (32.4)		
Above	1 (2.2)	1 (4.5)	2 (2.9)		
**Albumin**				t-test (56 df) = 1.77	0.082
Mean (SD)	3.3 (0.7)	2.9 (0.7)	3.1 (0.7)		
**Albumin classification** ^**1**^				Chisq. (2 df) = 0.64	0.727
Below normal	27 (58.7)	15 (68.2)	42 (61.8)		
Normal	12 (26.1)	4 (18.2)	16 (23.5)		
No information available	7 (15.2)	3 (13.6)	10 (14.7)		
**Serum iron**				Rank-Sum test	0.933
Median (IQR)	41.5 (26.0–82.0)	48 (31.0–64.0)	44 (27.0–72.0)		
**Total iron classification** ^**1**^				Chisq. (2 df) = 0.06	0.971
Below normal	16 (34.8)	7 (31.8)	23 (33.8)		
Normal	12 (26.1)	6 (27.3)	18 (26.5)		
No information available	18 (39.1)	9 (40.9)	27 (39.7)		
**Transferrin**				t-test (37 df) = 1.90	0.066
Mean (SD)	148.5 (40.2)	124.5 (33.2)	139.9 (39.2)		
**Transferrin classification** ^**1**^				Chisq. (1 df) = 0.21	0.644
Below normal	25 (54.3)	14 (63.6)	39 (57.4)		
No information available	21 (45.7)	8 (36.4)	29 (42.6)		
**Total iron binding capacity**				t-test (37 df) = 1.90	0.065
Mean(SD)	239.5 (50.4)	209.4 (41.4)	228.7 (49.0)		
**TIBC classification** ^**1**^				Chisq. (2 df) = 1.47	0.479
Below normal	14 (30.4)	10 (45.5)	24 (35.3)		
Normal	11 (23.9)	4 (18.2)	15 (22.1)		
No information available	21 (45.7)	8 (36.4)	29 (42.6)		
**Hemoglobin**				t-test (60 df) = 4.42	< 0.001
Mean(SD)	12.5 (1.8)	10.3 (1.8)	11.8 (2.0)		
**Anemia** ^**1**^				Fisher's exact test	0.005
Severe anemia	0 (0.0)	2 (9.1)	2 (2.9)		
Anemia	27 (58.7)	16 (72.7)	43 (63.2)		
Normal	16 (34.8)	1 (4.5)	17 (25.0)		
No information available	3 (6.5)	3 (13.6)	6 (8.8)		
**Hematocrit**				t-test (57 df) = 4.92	< 0.001
Mean (SD)	37.8 (4.7)	31.1 (5.1)	35.7 (5.7)		
**Hematocrit classification** ^**1**^				Chisq. (2 df) = 7.03	0.030
Below normal	24 (52.2)	18 (81.8)	42 (61.8)		
Normal	9 (19.6)	0 (0.0)	9 (13.2)		
No information available	13 (28.3)	4 (18.2)	17 (25.0)		
**Visceral and somatic protein nutrition** ^**1**^				Fisher's exact test	1.000
Malnutrition	44 (95.7)	21 (95.5)	65 (95.6)		
No information available	2 (4.3)	1 (4.5)	3 (4.4)		
**Serum zinc**				t-test (52 df) = 1.30	0.200
Mean (SD)	77.8 (20.8)	85.6 (19.4)	80.3 (20.5)		
**Zinc classification** ^**1**^				Chisq. (2 df) = 2.80	0.247
Below normal	15 (32.6)	3 (13.6)	18 (26.5)		
Normal	22 (47.8)	14 (63.6)	36 (52.9)		
No information available	9 (19.6)	5 (22.7)	14 (20.6)		
**Serum selenium**				Rank-Sum test	0.428
Median (IQR)	62.5 (47.2–74.8)	68 (53.1–76.7)	64 (47.7–76.1)		
**Selenium classification** ^**1**^				Fisher's exact test	0.910
Below normal	21 (45.7)	9 (40.9)	30 (44.1)		
Normal	8 (17.4)	3 (13.6)	11 (16.2)		
Above	5 (10.9)	3 (13.6)	8 (11.8)		
No information available	12 (26.1)	7 (31.8)	19 (27.9)		
**Serum vitamin A**				t-test (46 df) = 0.96	0.340
Mean (SD)	0.4 (0.2)	0.4 (0.2)	0.4 (0.2)		
**Retinol classification** ^**1**^				Fisher's exact test	0.593
Below normal	29 (63.0)	16 (72.7)	45 (66.2)		
Normal	3 (6.5)	0 (0.0)	3 (4.4)		
No information available	14 (30.4)	6 (27.3)	20 (29.4)		
**Serum vitamin E**				t-test (46 df) = 1.15	0.255
Mean (SD)	8.9 (4.5)	7.3 (4.3)	8.4 (4.4)		
**Tocopherol classification** ^**1**^				Fisher's exact test	0.346
Below normal	3 (6.5)	4 (18.2)	7 (10.3)		
Normal	29 (63.0)	12 (54.5)	41 (60.3)		
No information available	14 (30.4)	6 (27.3)	20 (29.4)		
**Micronutrient malnutrition** ^**1**^				Fisher's exact test	1.000
Malnutrition	38 (82.6)	18 (81.8)	56 (82.4)		
No information available	8 (17.4)	4 (18.2)	12 (17.6)		
**Total protein**				Rank-Sum test	0.021
Median (IQR)	7.7 (7.2–8.1)	8.5 (8.1–9.5)	8 (7.3–8.5)		
**Total protein classification** ^**1**^				Chisq. (2 df) = 9.03	0.011
Normal	14 (30.4)	3 (13.6)	17 (25.0)		
Above average	6 (13.0)	10 (45.5)	16 (23.5)		
No information available	26 (56.5)	9 (40.9)	35 (51.5)		
**Global nutrition** ^**1**^				Fisher's exact test	0.241
Malnutrition	30 (65.2)	18 (81.8)	48 (70.6)		
Nutrition	7 (15.2)	3 (13.6)	10 (14.7)		
No information available	9 (19.6)	1 (4.5)	10 (14.7)		
**Nutritional status** ^**1**^				Fisher's exact test	1.000
Malnutrition	45 (97.8)	22 (100.0)	67 (98.5)		
No information available	1 (2.2)	0 (0.0)	1 (1.5)		

^1^ = n (%)

Anemia = hemoglobin < 12g/dl; severe anemia = hemoglobin< 8g/dl; energy malnutrition = verified by BMI, MUAC and TSF; micronutrient malnutrition = verified by retinol, tocopherol, zinc, selenium and iron; protein malnutrition = verified by MAMC, albumin, TIBC; global malnutrition = verified by all the available nutritional parameters.

It is noteworthy that BMI measurements were less sensitive than TSF to detect energy malnutrition. BMI detected a quarter of patients with energy malnutrition at baseline and TSF detected more than two-thirds at baseline (Table 3).

**Fig 3:**
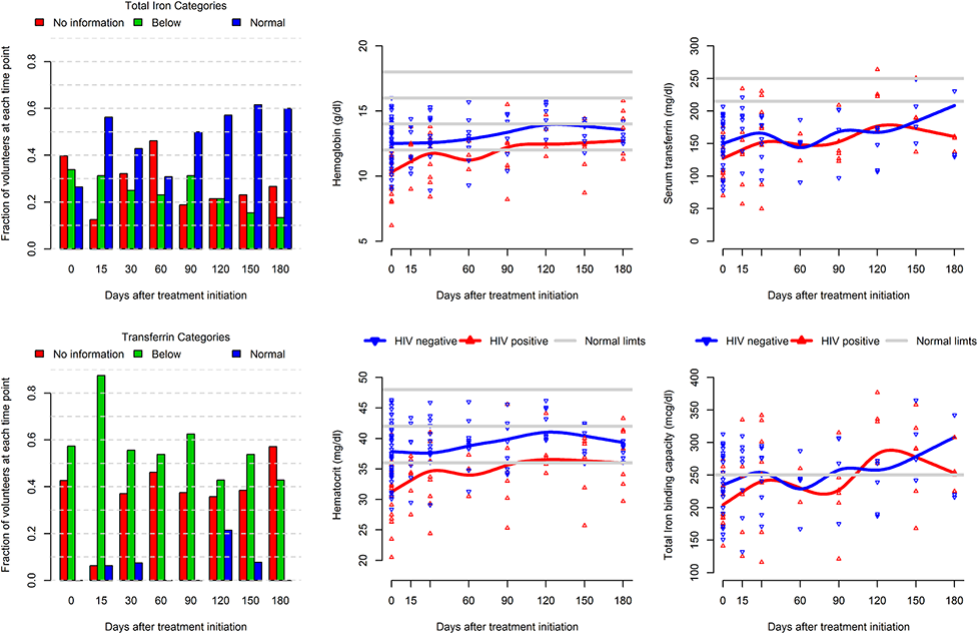
Body condition of iron related biomarkers in HIV-infected and non-infected patients during the tuberculosis treatment. Below = below the reference value.

A pattern of progressive increase over time was not observed for BMI, retinol and selenium. However, there was an apparent linear increase over time of the following parameters: weight, hemoglobin, hematocrit, iron fixation, transferrin, iron and tocopherol (Table 1 and Fig 3). The iron-related metabolism biomarkers showed a very slight trend of increase over time. However, this increase was not enough to reach the normal limits for most of the volunteers for all biomarkers but total iron (Fig 3). The remaining values appeared unstable and no linear trends could be established (Table 1).

The average transferrin and retinol levels were below the reference range and remained low until the end of the follow-up. In addition, patients exhibited a reduced ability to fix iron, and had low hematocrit and albumin levels (Table 1). The remaining parameters assessed were found to be below the reference range at the beginning of the study, and remained so to the end of the study, or the baseline mean values were within the normal reference range and remained stable through the study period (Table 1).

**Fig 4:**
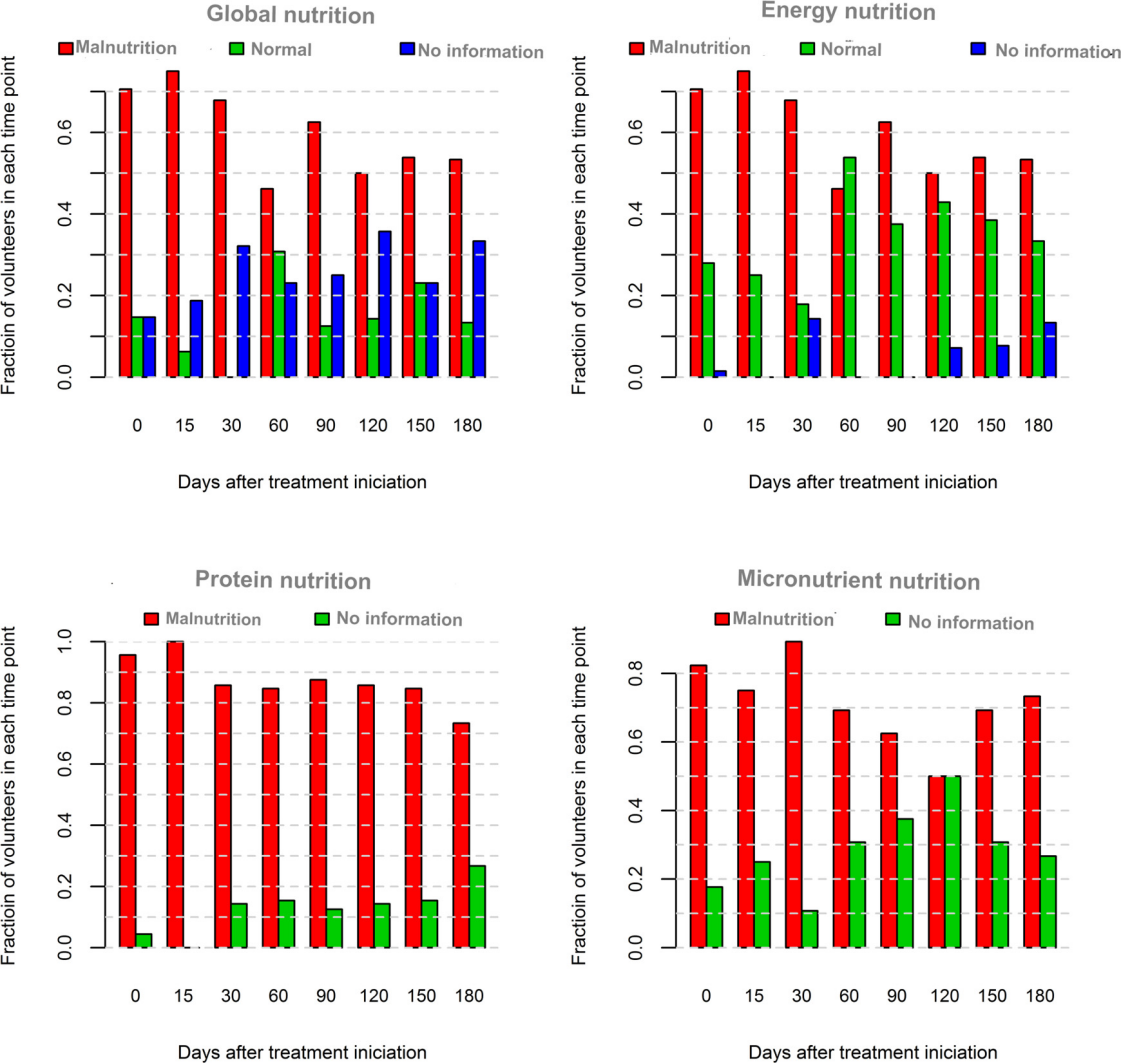
Fraction of different types of malnutrition of tuberculosis patients at each follow-up visit. Global malnutrition = BMI, TSF, MUAC, serum protein, albumin, transferrin and iron fixation below the reference value; energy malnutrition = BMI and TSF below the reference value; protein malnutrition = MAMC, serum protein, albumin, transferrin and iron fixation below the reference value; micronutrients malnutrition = serum biomarkers (iron, selenium, zinc, retinol and tocopherol) below the reference value.

The nutritional status did not show any apparent trend over time, except for the energy nutritional status. The energy nutritional status had an apparent increment after the second month of follow-up. However, a visually perceptible linear trend was not evident (Fig 4). Energy malnutrition is observed more often among individuals infected with HIV towards the end of the treatment course. These changes are not observed in other parameters used to assess nutritional status (Table 4).

**Table 4:** Nutritional status of HIV-infected and non-infected patients at the end of tuberculosis treatment.

Nutritional diagnosis	HIV- (%)	HIV+ (%)	Total
Energy malnutrition^1^	3 (42.9)	5 (71.4)	8 (57.1)
Protein malnutrition ^1^	5 (71.4)	5 (71.4)	10 (71.4)
Micronutrient malnutrition ^1^	5 (71.4)	6 (85.7)	11 (78.6)
Global malnutrition^1^	3 (42.9)	5 (71.4)	8 (57.1)
Nutritional status—malnutrition^1^	5 (71.4)	7 (100.0)	12 (85.7)
Selenium deficiency ^1^	3 (42.9)	2 (28.6)	5 (35.7)
Zinc deficiency ^1^	1 (14.3)	1 (14.3)	2 (14.3)
Tocopherol deficiency ^1^	1 (14.3)	0 (0.0)	1 (7.1)
Retinol deficiency^1^	5 (71.4)	5 (71.4)	10 (71.4)
Iron deficiency ^1^	2 (28.6)	0 (0.0)	2 (14.3)
Anemia ^1^	2 (28.6)	3(42.9)	5 (35.7)

1 = n (%); poor energy nutrition = verified by BMI, MUAC and TSF; specified malnutrition = checked for retinol, tocopherol, zinc, selenium and iron; protein malnutrition = verified by MAMC, albumin, TIBIC, transferrin; global malnutrition = checked using all available nutritional status parameters.

## Discussion

The main results of this study were: (a) all tuberculosis patients had malnutrition at baseline and, despite the provision of dietary counseling (following BMH’s and WHO’s recommendation), none of the patients recovered nutritional status during the six months of tuberculosis treatment; (b) the nutritional impairment was more intense at baseline in patients infected with HIV than in non-infected ones, regarding total protein and parameters related to iron metabolism.

Studies conducted in other countries showed impairment of nutritional status only in HIV-infected patients [25,26]. In the present study, there is evidence of malnutrition in both groups. These results may have been influenced by the use of anthropometric parameters other than BMI and the use of serum biomarkers to define malnutrition. This is an important issue, because the different concepts of malnutrition will lead to different abilities to detect it, probably leading to underestimation of its prevalence depending on the concept/method used. Therefore, a number of patients may miss the opportunity to receive appropriate diet counselling and nutritional supplementation, where malnutrition is underestimated.

The choice of a malnutrition concept that uses different nutritional parameters, besides the anthropometric, is a consequence of the fact that these additional parameters are considered important in the clinical progression of organic changes [23]. The concept that incorporates biochemical biomarkers comes from an awareness that several diseases can affect the nutritional status in a variety of ways. The different forms may include isolated changes or a combined deficiency of macro or micronutrients that are not always perceptible in anthropometric or semiological assessments. The risks of complications or morbid events associated with these diseases or nutritional status are different for each malnutrition type [23].

BMI is an anthropometric parameter for the monitoring of nutritional status. It has been used in clinical practice and at research settings to describe the nutritional status of study volunteers, and was adopted by the Brazilian Ministry of Health [5,20] and the World Health Organization [6] as an anthropometric index. Possibly, these entities utilize BMI for monitoring nutritional status due to its ease of use. Anthropometry is a technique that requires only easy-to-transport equipment, it is universal, and its application is inexpensive and non-invasive [27]. However, the criterion established by the FDA in 2013 [23] allows the inclusion of nutrition-related biomarkers results, including measurements of serum protein, iron and vitamins, body compartments and serum analysis of micronutrients. The results presented here in show that BMI is not sensitive enough to detect malnutrition. If patient evaluations were based on BMI alone, 60% to 70% of subjects included would be considered nourished.

The lack of sensitivity of BMI for the monitoring of nutritional status has already been described by other authors [28,29], who highlighted the possibility of an erroneous perception of recovered nutritional status. The specific deficits of protein and micronutrients cannot be identified using BMI. Following this premise, the MUAC measurement has been suggested as alternative anthropometric parameter for malnutrition diagnosis [30]. It was possible to verify in this study that the average MUAC remained below the normal limit in both groups in the whole observation period, and that although the TSF oscillated irregularly throughout the tuberculosis treatment, after the sixth month it remained below the normal limits, mainly in HIV-infected patients.

TSF is an anthropometric measure which is extremely sensitive to energy malnutrition [31,32]. There is evidence of TSF improvement during the tuberculosis treatment of patients not infected with HIV submitted to dietary supplementation [31,32]. However, the tuberculosis patients in this study indicated once more the importance of a careful and early analysis of additional parameters associated with anthropometry.

Regardless of whether the possibility that malnutrition had occurred prior to tuberculosis or HIV infection or not, there are several factors that may have influenced the patients’ food choices, or their access to more nutritionally complete foods, including: food taboos and beliefs [33], stigma associated with HIV and tuberculosis [34], presence or absence of family support [35–38], education [37–39]; and the high cost of food or low income [33,40,41]. The group studied here had an average of six years of education, income below a local minimum wage, and was aware of the stigma associated with the disease(s). Therefore, the combination of these factors may have negatively influenced their nutritional status, regardless of whether or not they have received dietary advice.

Energy malnutrition, and specially protein, iron and retinol deficiencies, could be the result of social issues, which were intensified by illness, and they remained after tuberculosis treatment was over. Since we did not have information on the nutritional status of the patients prior to tuberculosis and HIV infection, we are unable to confirm that the nutritional deficiencies were due to the illness or to their social condition prior to treatment. This assumption can be supported by published reports, which indicate that the brazilian population has moderate prevalence of malnutrition even without consumptive-associated diseases [42]. Consequently, it would be expected that a fraction of the treated patients would have some nutritional parameters improved, but their global nutritional status would not completely improve.

Another important point to be discussed is how long dietary counseling should be offered to be effective. This is a very challenging point, because if dietary counselling should be offered for a longer period than tuberculosis therapy, it would be necessary to retain patients for more than 6 months (in non-infected with HIV patients). Hospitals are not an adequate environment for healthy people and a more effective strategy is desired.

Surprisingly, vitamin A deficiency was identified in all of the volunteers throughout the treatment period, regardless of their HIV status. Studies conducted outside of Brazil showed a persistent vitamin A deficiency only in HIV-infected patients [43,44]. It is also known that hypovitaminosis affects 13 to 56% of the residents of Rio de Janeiro [42]. A retinol deficiency in 71.4% of tuberculosis patients not infected with HIV at the end of the treatment was unexpected, suggesting a persistent vitamin A deficiency on brazilians.

On the other hand, serum zinc and serum selenium deficiencies in these patients with this degree of malnutrition are not as intense as retinol deficiency, as expected in high oxidative stress conditions [45,46]. Selenium availability depends on protein levels to act as a cofactor for glutathione peroxidase [47,48] and Zinc is used as a catalyst and structural cofactor for about 3000 zinc-binding proteins [49], which are involved in the antioxidant process [50]. Therefore, the protein malnutrition, regardless of HIV status, determines the little consumption of both micronutrients for their antioxidant function.

We expected to find anemia in TB-HIV patients because both diseases are involved in different mechanisms that lead to anemia: the disease itself, antiretroviral therapy or blocked iron entry into the cell caused by *Mycobacterium tuberculosis* [51]. Anemia was observed in both groups but, at baseline, it was more intense in the HIV infected group.

As serum iron mean values did not change during tuberculosis treatment, the anemia and the impairment on the iron-related metabolism observation were possible because, besides total iron, other iron-related biomarkers were measured. In clinical practice, iron supplementation is neither used nor recommended in any guidelines, perhaps because iron deficiency and iron-related metabolism alterations are not so easily identified.

## Limitations

The main limitations of the study were the sample size and subject retention, both for the nutritional follow-up and for tuberculosis treatment. This lack of data, due to loss in follow-up, raises an uncertainty about the conclusions, as many outcomes are unknown. However, the prevalence of malnutrition observed at the end of follow-up was high, and selective loss in follow-up would be a problem only if this loss in follow-up is somehow related to full recovery of nutritional status. It is unlikely that this has happened as it would be necessary to observe a pattern in which preferably the subjects with the less impaired nutritional status are lost during follow-up. However, this pattern was not observed (data not shown). Some patients were discontinued because of severe malnutrition, to receive dietary supplement and others died. Also, this information could be interpreted as an absence of recovery of their nutritional status increasing the amount of patients with unfavorable outcomes. At the end, the lack of compliance with the follow-up could also be interpreted as a lack of effectiveness of the current recommendation. Another point is the lack of interventions to make patients feel “treated”, and the lack of financial support to allow them to choose the food they would like to eat.

## Conclusions

Patients infected with HIV had a worse condition at baseline, indicated by parameters related to protein and iron biomarkers. The nutritional status of all patients remained below the desired levels until the end of tuberculosis treatment. Adherence was a main challenge for medical treatment and nutritional counselling. Therefore, the evidence presented here does not support nutritional counselling as an effective recommendation.

## Supporting Information

S1 Dataset Information dataset file. (ZIP)
