# Correction: Nutritional Supplementation Is a Necessary Complement to Dietary Counseling among Tuberculosis and Tuberculosis-HIV Patients

**DOI:** 10.1371/journal.pone.0140737

**Published:** 2015-10-14

**Authors:** Adriana Costa Bacelo, Andrea Ramalho, Pedro Emmanuel Brasil, Cláudia dos Santos Cople-Rodrigues, Ingebourg Georg, Eliane Paiva, Sheila Vasques Leandro Argolo, Valeria Cavalcanti Rolla

The eighth author’s name is spelled incorrectly. The correct name is: Valeria Cavalcanti Rolla.
